# Editorial: AI, sensors and robotics in plant phenotyping and precision agriculture

**DOI:** 10.3389/fpls.2022.1064219

**Published:** 2022-11-23

**Authors:** Yongliang Qiao, João Valente, Daobilige Su, Zhao Zhang, Dongjian He

**Affiliations:** ^1^ Australian Centre for Field Robotics (ACFR), Faculty of Engineering, The University of Sydney, Sydney, NSW, Australia; ^2^ Information Technology Group, Wagenigen University & Research, Wageningen, Netherlands; ^3^ College of Engineering, China Agricultural University, Beijing, China; ^4^ Key Laboratory of Smart Agriculture System Integration, Ministry of Education, China Agricultural University, Beijing, China; ^5^ Key Laboratory of Agriculture Information Acquisition Technology, Ministry of Agriculture and Rural Affairs of China, China Agricultural University, Beijing, China; ^6^ College of Mechanical and Electronic Engineering, Northwest A&F University, Yangling, Shaanxi, China

**Keywords:** artificial intelligence, plant phenotyping, precision agriculture, smart sensors, agricultural robotics, UAV

## Introduction

Plants and their production play an important role in retaining the sustainability for the natural ecosystem and human beings’ food security. With the increasing global population, rapid urbanization and climate change, how to improve plant protection levels, increase plant breeding speed and make sure the agricultural planting in a sustainable and low-carbon dioxide manner becomes challenging. One way to address this issue is to develop the technology of plant phenotyping and precision agriculture (Costa et al.). Plant phenotyping and precision agriculture as information- and technology-based approaches, could evaluate a large amount of plants and provide effective information to production management. Plant phenotyping assesses complex plant traits such as plant morphology, plant stress, crop yield, plant physiological, anatomical traits, and genotype performance under distinct environmental conditions. Precision agriculture is aimed at examining spatial heterogeneities within crop stands based on the spatial and temporal variability in crop and soil factors within a field (Stafford, 2000 and Patrício and Rieder, 2018). High-throughput phenotyping in precision agriculture is helpful to improve management practices, and efficient phenotyping in the field also reduces the invested resources (e.g., fertilizer, water, pesticide).

**Figure 1:**
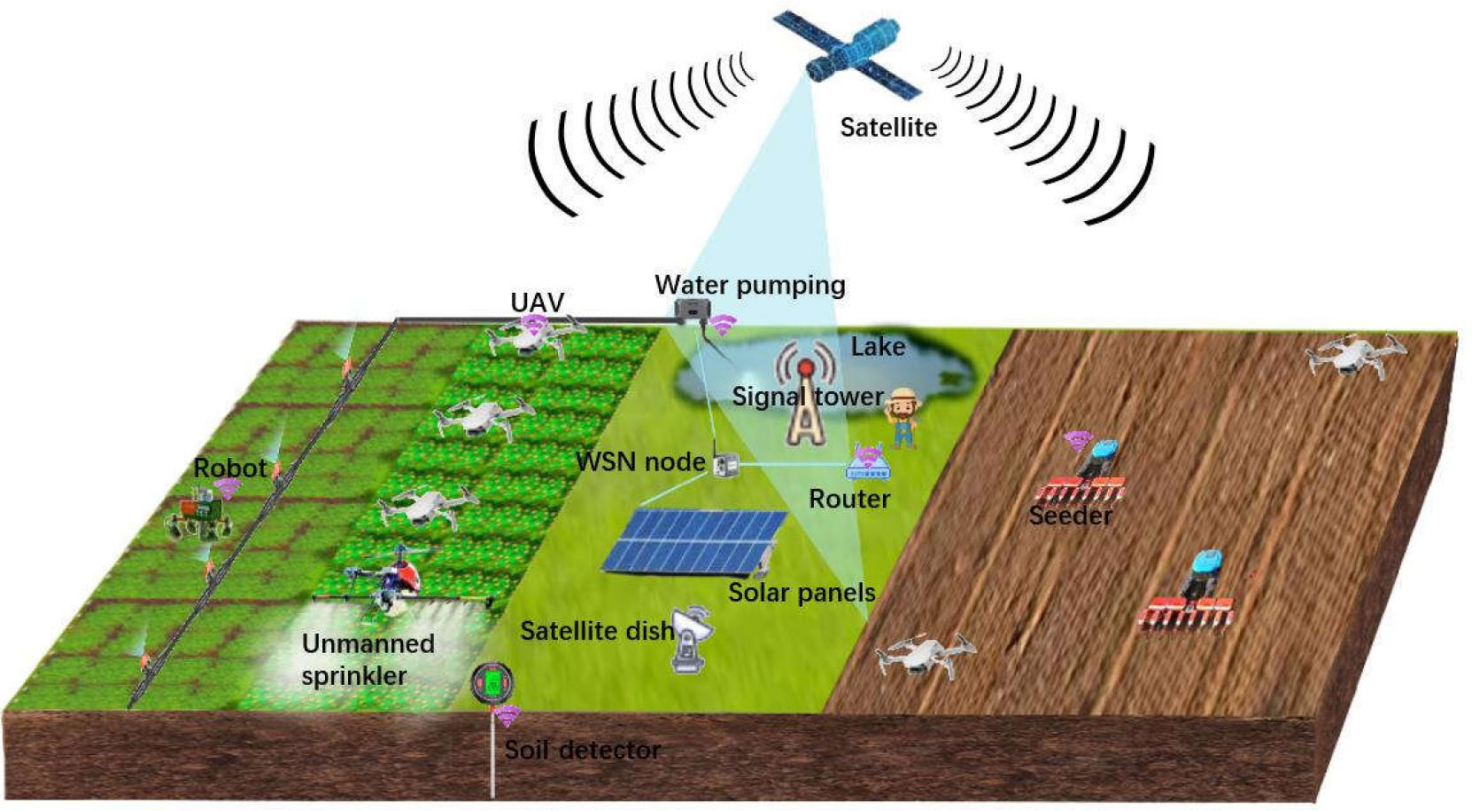
AI, sensors and robotics in plant phenotyping and precision agriculture.

In recent years, cutting-edge technologies for plant phenotyping and precision agriculture are fundamental to improve the productivity and sustainability of plant production systems (Narvaez et al., 2017). Especially, the development of Artificial Intelligence (AI), smart sensors and robotics provides a non-invasive manner for assessing complex traits in plants (as shown in **Figure 1**), measuring plant-physiological parameters, diagnosing plant diseases, predicting the yield and performance of plants at various organizational scales (Purcell and Neubauer, 2023).

The comprehensive plant phenotyping emerges from the dynamic and local interaction of phenotypes with the spatially and temporally dynamic environment above and below ground, while assessing complex plant traits such as growth, tolerance, resistance, physiology, ecology, plant stress and yield, which benefits the farmers and plant breeders to identify phenotyping parameters and select desirable genotypes that provide effective information to make informed agricultural production management decisions (Li et al., 2014). By assessing complex plant traits (e.g., growth, development, resistance, physiology, ecology), high yielding and stress-tolerance crop varieties adapt to future climate conditions and resistant to pests and diseases, produce enough food, feed, fiber, and fine chemicals in next century to meet the needs of a growing population worldwide (Abbasi et al., 2022).

## Plant phenotyping

Plant monitoring and phenotyping can reflect many valuable parameters and effective information for optimizing agricultural production management in smart farming. Traditional manual based methods rely on experienced farmers, which is of low-accuracy and poor efficiency. Nowadays, a range of sensors (various RGB, multi-and hyperspectral cameras, 3D-sensors, etc.) and platforms have been used to realize real-time, rapid, and efficient plant phenotyping. According to different perception principles, these sensors mainly have ground feature spectrometers, spectral imaging sensors, and other imaging spectrometers.

Qin et al. proposed a real-time and low-cost Ag-YOLO model for crop monitoring and crop spraying, which achieved 0.92 F1-score with a speed of 36.5 frames per second (fps) on Intel Neural Compute Stick 2 (NCS2). Liu et al. proposed a portable wild phenotyping system based on segmentation results from DeepLabV3+ model to obtain 45 traits, including 15 plant traits, 25 leaf traits and 5 stem traits. The proposed system provides a solution for maize phenotyping in the field and benefit crop breeding. Lu et al. proposed a soybean yield in-field prediction method based on bean pods and leaves image recognition using a deep learning algorithm combined with a generalized regression neural network (GRNN). According to the experiments, the soybean yield of each planter was obtained by accumulating the weight of all soybean pod types and the average accuracy was up to 97.43%.

In addition, an identification model YOLO-VOLO-LS was constructed for hydroponic lettuce grown in a greenhouse under the conditions of different growth periods (Zhang and Li). By combining the respective advantages of the target detection mechanism and the classification mechanism, a nearly 100% of the lettuce classification effect in the growth stages of days 1, 6, 12, 18, 24, and 30 were achieved. Wang et al. proposed a lightweight model based on the improved You Only Look Once version 4 (YOLOv4) to detect dense plums in orchards, which achieved 86.34% detection accuracy.

For obtaining image-based phenotypic information of wheat traits for spike morphology analysis and yield estimation, Zhang et al. collected wheat images from fields and proposed an optimized hybrid task cascade model for automatic dense wheat spike segmentation. Experimental results showed that they achieved an average precision (AP) of 90.7%, and an accuracy of 99.29%.for wheat spike counting. Qiu et al. processed the color images of the spike in YCbCr color space and then utilized Faster R-CNN to detect the spikelets. Testing results showed that the root mean squared errors between the automatic and manual counted spikelets for four wheat lines were 0.62. Qi et al. proposed a novel tea chrysanthemum–generative adversarial network (TC-GAN) for tea chrysanthemum detection, which achieved an optimal average precision (AP) of 90.09%.

Nitrate nitrogen plays an important role during crop growth, and the operation of Increasing N fertilizer dosage and application is usually one of the essential ways to boost crop productivity. Su et al. proposed an ISE system combined with a temperature sensor and a pH electrode to automatically measure the concentrations of Nitrate nitrogen.

As sugar being the energy source of plants and plays an important role in plant growth and development, Liu et al. developed an enzyme-free electrochemical sensor for *in situ* detection of reducing sugar, which demonstrated that the COOH-GR–COOH-MWNT–AuNP-modified electrode exhibited a good catalysis behavior. To investigate the study effect of vegetation distribution on mean flow velocity and turbulence characteristics in a channel, Wang et al. constructed a flow velocity distribution model to study the microscopic mechanism of the flow velocity distribution in the upper layer of vegetation, which provides a solution for flow measurement in the ecological channel.

Hyperspectral imaging is advantageous in delivering reliable and comprehensive analysis of characteristics or properties of plant materials, which is a powerful modality for measuring spectral and spatial information of samples simultaneously (Lu et al., 2020). Lu et al. classified industrial hemp cultivars, growth stages, and plant organs (leaves vs. flowers) using hyperspectral imaging technology. Based on regularized linear discriminant analysis, an accuracy of up to 99.6% was achieved in differentiating the five hemp cultivars. Liu et al. designed a near-infrared (NIR) phenotypic sensor for predicting wheat gain quality, and the R2 of the relative diffuse reflectance (RDR) of all four wavelengths of the phenotypic sensor and the reflectance of the diffusion fabrics were higher than 0.99.

## Plant diseases detection

Diseases are the main causal factors affecting crop growth and yield. Reliable and timely plant disease detection is important for plant protection activities, field crop growth and plant breeding. AI and computer vision based diagnosis and detection of plant diseases must consider that the occurrence of plant disease depends on specific environmental factors and diseases often exhibit a heterogeneous distribution in fields (Mahlein, 2016).

Wang et al. proposed a YOLOv3-tiny-IRB algorithm to improve the detection accuracy of tomato diseases and pests under conditions of occlusion and overlapping in real natural environment, which achieved the mean average precision (mAP) of 98.3, 92.1, and 90.2%, respectively under three conditions: (a) deep separation, (b) debris occlusion, and (c) leaves overlapping. Zhang et al. proposed YOLOv5-CA based GDM detection approach for grape downy mildew disease detection, and the experimental results show that the proposed YOLOv5-CA achieved a detection precision of 85.59%, a recall of 83.70%, and a mAP@0.5 of 89.55%, which are superior to the popular methods, including Faster R-CNN, YOLOv3, and YOLOv5.

Chen et al. proposed 2D histogram Otsu based approach for segmenting maize foliar disease images, the experimental results indicated that the method effectively improved the segmentation of the three maize disease spot images and could obtain more apparent disease spot areas. Zhang et al. extracted handcrafted and deep features from the color image and color-infrared (CIR) image, and the DFs coupled with parallel feature fusion resulted in diagnosis accuracies of over 70%.

## Robotics and UAVs in smart farming

Robotics and UAVs have shown great efficiency and effectiveness in the agriculture field. In recent years, many agricultural related robotics and UAVs have been designed and developed to manage crops, plants, livestock and fishes (Qiao et al., 2019; Su et al., 2021; Li et al., 2022 and Du et al., 2022). Based on the Simultaneous Localization and Mapping (SLAM), place recognition and autonomous navigation, robots or UAVs can autonomously drive and perform actions such as harvesting, picking and trimming.

In robotic precision spray of vegetables, accurate and reliable detection and tracking of every vegetable is of utmost importance. Hu et al. proposed LettuceMOT, a multiple object tracking (MOT) method to correlate these re-appeared vegetables with their previous identities. The experimental results show that LettuceMOT outperformed existing state-of-the-art MOT methods (e.g., ByteTrack, FairMOT, TraDeS and SORT).

To achieve the rapid harvesting of table grapes planted with a standard trellis in the grape industry, Jiang et al. carried out a dual-arm high-speed grape-harvesting robot to improve low picking efficiency. Robotic arm and camera view analysis of the workspace harvesting process was performed using MATLAB, and it can be concluded that the structural design of this robot meets the grape harvesting requirements with a standard trellis. The field performance test verifies that the average harvesting cycle of the robot with both arms reached 9 s/bunch, and the success rate of bunch identification and harvesting success rate reached 88% and 83%, respectively, which were significantly better than those of existing harvesting robots worldwide.

In terms of agricultural navigation technologies, Xie et al. proposed the miniaturization scheme of zooming detection arc based on variable central angle and established the adjustment equation of the detection distance of photoelectric switches at each position, a small integrated photoelectric arc array navigation sensor with a cost of about $65 is developed using an embedded microcontroller. However, there is still a problem of external noise and other factors causing the failure of the navigation system. To solve this problem, Lv et al. proposed an agricultural scene-based multi-sensor fusion method *via* a loosely coupled extended Kalman filter algorithm to reduce interference from external environment. Specifically, the proposed method fuses inertial measurement unit (IMU), robot odometer (ODOM), global navigation and positioning system (GPS), and visual inertial odometry (VIO), and uses visualization tools to simulate and analyze the robot trajectory and error. In experiments, the high accuracy and the robustness of the proposed algorithm were verified when sensors fail. The experimental results show that the proposed algorithm has better accuracy and robustness on the agricultural dataset than other algorithms.

For phenotypic feature detection in the study of automatic trimming, Tang et al. optimized and designed a long-belt finger-clip precision seed metering device, which includes a diffuse reflection photoelectric sensor and rectangular optical fiber sensor to monitor the number of corn seeds in the seeding process. To automatically trim hedges, Zhang et al. proposed a binocular vision-based shape reconstruction and measurement system, based on stereo correcting algorithm and an improved semi-global block matching (SGBM) algorithm The center coordinate and radius of the spherical hedges can be measured. The outdoor test shows that the average error and average relative error of spherical hedges radius by the proposed system are 4.02 mm and 0.44%, respectively. The average location deviation of the center coordinate of spherical hedges is 18.29 mm.

Fu et al. quantified the forces on the stalks caused by root anchorage in corn harvesting, and a root force measurement system was designed and applied in this study. The bending moment and torsional moment on the upright and lodged corn stalks were measured in corn harvesting. By analyzing the bending moment curves on the lodged corn stalks, it was proposed that for the harvesting of corn lodged in the forward, reverse, and lateral direction, the corresponding harvester header improvement suggestions are enlarging the size of pins on the gathering chains, reducing the speed of gathering chains, and lengthening the snouts with a sleeker surface, respectively. This study provides base data for the root anchorage effect on lodged corn and provides references for the improved design of the corn harvester header.

On the other hand, UAV based platform are also popular in the precision agriculture and plant phenotyping applications because their large cover range and higher data scanning speed. In addition, UAVs can fly automatically with less human intervention during data collection. Zhaosheng et al. improved wheat ears identification performance in a field environment using improved YOLOX-m model. To develop a data processing pipeline for performing fast and accurate pre-harvest yield predictions of cotton breeding fields using aerial image, Rodriguez-Sanchez et al. used a Support Vector Machine (SVM) classifier with four selected features to identify the cotton pixels present in each plot image, which achieved an accuracy of 89%, a precision of 86%, a recall of 75%, and an F1-score of 80% at recognizing cotton pixels. This study demonstrates that aerial imagery with machine learning techniques can be a reliable, efficient, and effective tool for pre-harvest cotton yield prediction. Krul et al., 2021 studied the feasibility to apply UAV for indoor farming monitoring and control. The performance of different state-of-the-art visual simultaneous localization and mapping (VSLAM) algorithms with a small and low-cost UAV was assessed. The authors found that ORB-SLAM was the algorithm that perform best in such an environment. Tests in the farming facilities where performed and different maps were generated.

Finally, agricultural management could also benefit from the collaboration between aerial and ground robotic systems. The aerial robotics could survey a field using different types of sensors and payloads. Moreover, it could provide the ground robot a detailed map with specific positions where the ground robot need to inspect further or perform some action with an actuator. Conesa-Muñoz et al., 2016 proposed a multi-robot system to reduce the amount of herbicide during site-specific treatments. The combination of aerial-ground robotics systems allows to reach a 97% spray accuracy and a mean deviation lower than 7cm. Zhang et al., 2022 investigated the spatial-variability of orchards flower blossom from an aerial and ground perspective. Several point clouds where acquired in a commercial orchard (Elstar) field using a UAV and a ground vehicles. The feasibility of combing data from both platforms to assess flowering intensity at the tree-level, was demonstrated, yielded R^2^>0.7 and RMSE lower than 20.

## Conclusions

Plant phenotyping and precision agriculture is becoming a very important topic for future agriculture. The increasing population and climate change push us to take actions to plant crops against pests, diseases, and harsh environments (e.g. lack of nutrients, water, fertilizers or light). The new technologies such as AI, sensors and robotics enables farmers to take a data-driven approach to collect and analyze data to monitor the real-time status of the plans and crops to improve production yield quality. For precision agriculture, the grand challenges lie in identification of cheap, robust, easy-to-use, rapid and automated phenotyping methods that can feed into Decision Support System. In addition, the field environment will provide challenges in sometimes rapidly varying light conditions, wind and temperature, as well as combinations of multiple stresses. Despite all these challenges, automated and systematic stress detection by field-phenotyping holds great promise to accelerate Integrated pest management where on-farm live monitoring of stress and disease are key factors to reduce the reliance on pesticides.

In the future, the integration of automated data collection and analysis, AI algorithms, robotics and decision support systems will bring unmanned farming to our lives. Moreover, the ground-level or aerial-level robotic systems will also have a major role in plant phenotyping and precision agriculture, for monitoring, disease control and harvesting.

## Author contributions

YQ: investigation, methodology, writing-original draft, and editing. JV, DS, ZZ, and DH: writing-review and editing. All authors contributed to the article and approved the submitted version

## Conflict of interest

The authors declare that the research was conducted in the absence of any commercial or financial relationships that could be construed as a potential conflict of interest.

## Publisher’s note

All claims expressed in this article are solely those of the authors and do not necessarily represent those of their affiliated organizations, or those of the publisher, the editors and the reviewers. Any product that may be evaluated in this article, or claim that may be made by its manufacturer, is not guaranteed or endorsed by the publisher.
